# Identifying network biomarkers based on protein-protein interactions and expression data

**DOI:** 10.1186/1755-8794-8-S2-S11

**Published:** 2015-05-29

**Authors:** Jingxue Xin, Xianwen Ren, Luonan Chen, Yong Wang

**Affiliations:** 1 Academy of Mathematics and Systems Science, Chinese Academy of Sciences, Beijing 100190, China; 2 National Center for Mathematics and Interdisciplinary Sciences, Chinese Academy of Sciences, Beijing 100190, China; 3 MOH Key Laboratory of Systems Biology of Pathogens, Institute of Pathogen Biology, Chinese Academy of Medical Sciences and Peking Union Medical College, Beijing 100730, China; 4 Key Laboratory of Systems Biology, Shanghai Institutes for Biological Sciences, Chinese Academy of Sciences, Shanghai 200233, China

## Abstract

Identifying effective biomarkers to battle complex diseases is an important but challenging task in biomedical research today. Molecular data of complex diseases is increasingly abundant due to the rapid advance of high throughput technologies. However, a great gap remains in identifying the massive molecular data to phenotypic changes, in particular, at a network level, i.e., a novel method for identifying network biomarkers is in pressing need to accurately classify and diagnose diseases from molecular data and shed light on the mechanisms of disease pathogenesis. Rather than seeking differential genes at an individual-molecule level, here we propose a novel method for identifying network biomarkers based on protein-protein interaction affinity (PPIA), which identify the differential interactions at a network level. Specifically, we firstly define PPIAs by estimating the concentrations of protein complexes based on the law of mass action upon gene expression data. Then we select a small and non-redundant group of protein-protein interactions and single proteins according to the PPIAs, that maximizes the discerning ability of cases from controls. This method is mathematically formulated as a linear programming, which can be efficiently solved and guarantees a globally optimal solution. Extensive results on experimental data in breast cancer demonstrate the effectiveness and efficiency of the proposed method for identifying network biomarkers, which not only can accurately distinguish the phenotypes but also provides significant biological insights at a network or pathway level. In addition, our method provides a new way to integrate static protein-protein interaction information with dynamical gene expression data.

## Introduction

The rapid advance of high-throughput technologies opens a new way for biomarker identification, which is an important but challenging task in biomedical research. By exploring mRNA and protein expression profiling, many sophisticated methods have been developed to identify biomarkers for classifying and diagnosing diseases and their severities. However, most conventional biomarker discovery methods mainly rely on expression measurements of individual molecules with less focus on their associations or interactions. A typical method is to select differentially expressed genes from normal and disease samples with gene expression profiling data as candidate biomarkers, and then identifies the gene biomarkers by constructing an expression-based classifier using machine learning or pattern recognition techniques. In such a way, a number of biomarkers have been successfully identified, but the methods failed to detect effective biomarkers with a high accuracy across many datasets. Major reasons for such diagnostic failure lie in (1) the heterogeneous nature of complex diseases whose expressions may vary considerably from patient to patient [1] and (2) small sample sizes that may be inadequate for consistently capturing individual disease genes [2]. Recently, an effective method, the so-called network biomarker [2], has been developed, and it can diagnose the disease state in an accurate manner by combining the information a of network (e.g., protein-protein interaction data) with gene expression measurements [1,3]. Studies on clinical samples have shown the success of this methodology in diagnosis of metastatic breast cancer by overlaying a patient's expression profile onto the human protein-protein interaction map [1].

Generally, it is difficult to measure the activities of protein-protein interactions in a cell. One way is to approximate their activities by using the available gene expression data. For example, co-expression of a pair of proteins is a simple method to assess how active a protein-protein interaction is in certain conditions. The Pearson correlation coefficient (PCC) as well as other related quantitative criteria are widely used to measure gene co-expression network [4,5]. By exploring network information for biomarker identification, recently Zhang et al. [6] defined a vector representation in edge space based on the decomposed PCC to find gene pairs as edge biomarkers, which demonstrates the ability and potential of network information. In particular, their results show that many edge biomarkers (i.e., protein or gene pairs) can distinguish normal and disease samples in high accuracy but their differential expressions are actually not significant. In other words, as individual molecules, these genes in edge biomarkers have no distinguishable power but their correlations can well classify the samples with distinct phenotypes. In this work, we take a similar way to approximate PPI activity, which can be expressed by the law of mass action.

The biological data are usually high-dimensional with a large number of variables but a small number of samples. Supposing that there are 10,000 genes, the possible number of edges or molecular pairs will be 10,000^2^, whose computation may suffer from the serious problem of the "curse of dimensionality". Thus, dimension reduction and feature selection methods are in pressing need. Feature selection is a key issue in statistics and machine learning. Traditional statistical methods, such as Akaike information criterion (AIC) and Bayesian information criterion (BIC), penalize the number of parameters in the model, but they need to screen all the subsets of the parameters, which is infeasible in terms of computational complexity in high-dimensional data. LASSO [7] uses L_1 _penalty in the linear regression form and makes the estimate sparse. It only deals with continuous response variables and sometimes over-selects. Support vector machine trains a set of support vectors on the margin of different classes to linearly separate the samples in feature space [8,9]. In contrast, EllipsoidFN [10] and LPFS [11] are novel feature selection methods based on linear programming model with fast convergence to the optimal solution. Particularly, sophisticated algorithms for linear programming allow efficient feature selection from ultra-high dimensional data.

In this paper, we propose a novel method to estimate the protein-protein interaction affinity (PPIA) from gene expression data based on the law of mass action [12], whose information is further used to identify a set of interactions and gene-nodes as network biomarkers to diagnose diseases [10]. We name this new method PPIA + ellipsoidFN. Different from the traditional gene-node based method [10] or DEG + ellipsoidFN method, our PPIA + ellipsoidFN method considers the network information from protein-protein interactions in biological processes, enhances the interpretability of the biomarkers, and thus can achieve accurate diagnosis on the phenotypes of diseases. Computational results on three breast cancer datasets demonstrate that our method can identify a set of network biomarkers with a low redundancy but high accuracy.

## Method

### Overview of the new network biomarker identification method

**Figure 1:**
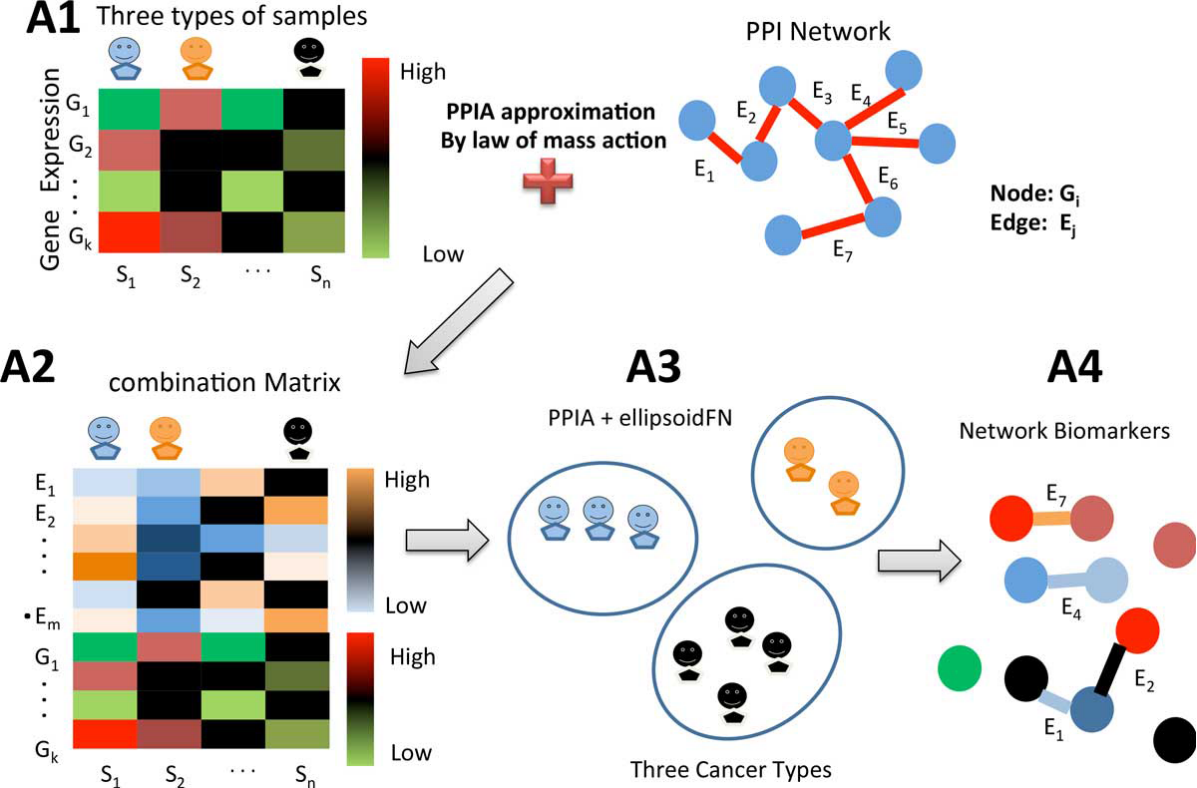
**The workflow for PPIA + ellipsoidFN method**. (A1) Gene expression data for cancer (rows are genes and columns are samples with multiple different types) and human protein-protein interaction network. (A2) Combining gene expression data with human PPI network by product approximation, where each protein-protein interaction affinity E_i _can be computed based on the law of mass action. The rows are edges of PPI or gene nodes and the columns are samples. (A3) By the PPIA+ellipsoidFN method, different cancer types or normal and disease case can be represented by different ellipsoids, and the distances among these ellipsoids are maximized. (A4) PPIA network biomarkers are identified by the PPIA+ellipsoidFN method to classify different phenotypes.

We propose a novel method to estimate the protein-protein interaction affinity from gene expression data and further to identify a set of protein-protein interactions and single proteins as network biomarkers for diagnosing diseases. Firstly PPIA is approximated by the law of mass action. Then we construct a linear programming model to identify a set of PPIAs and single proteins as network biomarkers, where theoretically each class or cluster of samples (i.e., case or control samples) is represented by an ellipsoid. This optimization model aims to select minimal number of PPIs and proteins to maximize the distance between different ellipsoids. More precisely, this process can find a minimal set of PPIs and proteins as network biomarkers, which divide normal and disease samples or different cancer types as distinct as possible. Figure 1 shows the workflow of the whole PPIA + ellipsoidFN method.

### Approximating the protein-protein interaction affinity by law of mass action

In 1864, Guldberg and Waage [12] suggested that in a reaction such as:

$$A+B\overset{}{⇆}A^{\prime }+B^{\prime }$$

The "chemical affinity" between A and B not only depends on the chemical nature of the reactants, but also depends on the amount of each reactant in a reaction mixture. Thus the Law of Mass Action was first stated as follows:

When two reactants, A and B, react together at a given temperature in a "substitution reaction", the affinity, or chemical force between them, is proportional to the active masses [A] and [B], each raised to a particular power. The chemical force is assumed to be directly proportional to the product of the active masses of the reactants, thus the affinity equation can be rewritten as:

$$affinity=\alpha \left[A\right]\left[B\right].$$

The proportionality constant was called an affinity constant, α.

As a result we can approximate the activities of protein complexes according to the elementary reactions and the gene members of the protein complex. For example, protein P_1 _interacts with protein P_2 _to form a protein complex [P_1_P_2_] and perform a biological function. In our study, we need to assess the abundance of protein complex [P_1_P_2_] formed by the protein-protein interaction between protein P_1 _and protein P_2_. By the law of mass action demonstrated above, we have

$$\left[P_{1}P_{2}\right]=\alpha \left[P_{1}\right]\left[P_{2}\right].$$

Let x_1 _and x_2 _represent the mRNA expression levels of the corresponding proteins P_1 _and P_2_. After the translation process, the concentrations [P_1_] and [P_2_] of individual proteins P_1 _and P_2 _before various modifications and reactions, are proportional to their mRNA expression levels, i.e., [*P*_1_] ≈ *x*_1_, [*P*_2_] ≈ *x*_2_. In our computation, we assumed that the affinity constants are the same for all the protein-protein interactions and set it to one. Therefore, we can use the following equation to estimate protein-protein interaction affinity for each interaction for any pair of proteins P_1 _and P_2_:

$$\left[P_{1}P_{2}\right]=x_{1}x_{2}.$$

### Optimization model to identify a minimal set of protein interactions for classifying cancers and normal samples

Given a gene expression dataset *X*_*m*×*n*_, in which the expression of n genes is measured for m samples and *x_ij _*denotes the expression level of gene or protein j in sample i. For a single protein-protein interaction, we can approximate the protein-protein interaction affinity by the law of mass action. As a result, a protein-protein interaction affinity matrix can be derived as *A*_*m*×*q*_, in which the affinity of q protein-protein interactions is calculated for m samples. *a_ij _*= *x_iu_x_iv _*denotes the affinity level of protein-protein interaction j (between proteins *u *and *v*) in sample i. We set *w_i_, i *= 1,..., *q*, denoting the weight for each protein-protein interaction to be selected as an edge biomarker. Similarly, *w_i_, i *= *q *+ 1,...,*q *+ *n *are weights for each gene to be selected as node biomarker. These two part of biomarkers are combined to form the network biomarker set. Supposing in total that there are c classes of samples, the formulation of our method can be described as follows:

(1) $$\text{min}\overset{q}{\underset{i=1}{\sum }}w_{i}+\lambda \overset{q+n}{\underset{i=q+1}{\sum }}w_{i}+\alpha \overset{c}{\underset{i=1}{\sum }}\left(z_{1}^{i}-z_{2}^{i}\right)+C\overset{m}{\underset{i=1}{\sum }}\overset{c}{\underset{j=1}{\sum }}\xi_{ij}$$

Subject to

(2) $$\overset{q}{\underset{i=1}{\sum }}w_{i}{\left(a_{ji}-a_{i}^{k}\right)}^{2}+\overset{q+n}{\underset{i=q+1}{\sum }}w_{i}{\left(x_{ji}-x_{i}^{k}\right)}^{2}\leq z_{1}^{k}+\xi_{jk}\;\;\text{for }j\in I^{k},k\in \left\{1\cdots c\right\}$$

(3) $$\overset{q}{\underset{i=1}{\sum }}w_{i}{\left(a_{ji}-a_{i}^{k}\right)}^{2}+\overset{q+n}{\underset{i=q+1}{\sum }}w_{i}{\left(x_{ji}-x_{i}^{k}\right)}^{2}\geq z_{2}^{k}-\xi_{jk}\;\;\text{for }j\notin I^{k},k\in \left\{1\cdots c\right\}$$

(4) $$0\leq z_{1}^{k}\leq z_{2}^{k}\;\;\text{for k}\in \left\{1\cdots c\right\}$$

(5) $$0\leq w_{i}\leq 1\;\;\text{for }i\in \left\{1\cdots q+n\right\}$$

(6) $$\xi_{ij}\geq 0\;\;\text{for }i\in \left\{1\cdots m\right\}\text{ and }j\in \left\{1\cdots c\right\}$$

Where $a_{i}^{k}=\sum_{j\in I^{k}}x_{ju}x_{jv}/m_{k}$ is the average/median affinity level of protein-protein interaction *i *(between proteins *u *and *v*) in class k for all samples. *I^k ^*is the set of samples belonging to class k with total number *m_k_*. Similarly $x_{i}^{k}$ denotes the average/median expression level of gene *i *in class k for all samples. $z_{1}^{k}$ and $z_{2}^{k}$ are variables defining the inner and outer radius of the ellipsoid representing class k. *ξ_ij _*are slack variables to tolerate the data errors. Equation (1) is the objective function for the optimization problem, which consists of three terms. $\overset{q}{\underset{i=1}{\sum }}w_{i}$ denotes the weight summation of selected protein-protein interactions. By minimizing it, we aim to select the smallest number of protein-protein interactions as biomarkers to enhance the interpretability. In computational experiments we set 10^-4 ^as a threshold. The weight larger than the threshold for each protein-protein interaction is selected as a network biomarker. The second term $\overset{c}{\underset{i=1}{\sum }}\left(z_{1}^{i}-z_{2}^{i}\right)$ is minimized to enlarge the difference of inner and outer radius of ellipsoid for clear separation of each class. The third term $\overset{m}{\underset{i=1}{\sum }}\overset{c}{\underset{j=1}{\sum }}\xi_{ij}$ denotes the total classification error for all the samples, which is minimized to achieve high classification accuracy. Here λ, α, and C are three parameters introduced to balance the above three goals and unify them into a single objective function. In computational experiments we assume edges and nodes (PPIs and single proteins) have the same importance, so we introduce the square of the expression value of each gene, comparable to the product-form approximation of PPIA, to substitute the original gene expression level. Then one parameter λ can be set to one. We can reduce the number of the parameters in this simple way. The remaining two parameters α and C are determined by grid search with the optimal predicting accuracy. For α we tested 0.01, 0.02, 0.1, 0.5, 1, 5, 10 and for C we tried 0.1, 1, 10, 100 and 1,000. Equation (2) implies the assumption (1), i.e., samples from the same cancer type are enclosed by one ellipsoid, which minimizes the distance of a sample from its class center. Equation (3) implies the assumption (2), i.e., every sample from the other cancer locates outside of the ellipsoid representing the current cancer. The divergence of one cancer sample from another or normal samples is measured by the weighted sum of the divergence of protein-protein interaction affinities and proteins such that heterogeneity is modeled. The goal is to identify a minimal set of protein-protein interactions that maximize the distances between ellipsoids. We used the quadratic function in constraints (2) and (3). Other nonnegative functional forms, e.g., the absolute values, can also be applied in a similar way.

### Datasets and evaluation scores

The gene expression datasets for evaluation include NCBI GEO database with accession number GSE10797 [13], GSE7904 [14], and GSE18229 [15]. The former two datasets have normal and disease cases, and the latter has five subtypes of breast cancer. Also the human protein-protein (PPI) interaction data was downloaded from HPRD database. This dataset consists of 39,240 protein-protein interactions. After deleting interactions with missing values and self-loops, 36,888 interactions left.

We compared our method with node-based ellipsoidFN [10] and t-test in accuracy, redundancy score, and biological functions for both two-class case and multiple-class case. The scores we used to evaluate the performance of these three methods are predicting accuracy and redundancy score. Predicting accuracy is computed based on leave-one-out or 10 fold cross-validation using biomarkers identified in each method. We can compute Pearson correlation coefficients for pairwise proteins, and the redundancy score for a set of proteins is defined as the mean PCC' for every pair of them in the set, where PCC' is absolute value of PCC. Given a protein set {p_1_, p_2, ..., _p_n _}, the redundancy score is defined as follows,

$$redundancy\;score\;=\frac{\underset{i\neq j}{\sum }\left|cor\left(p_{i},p_{j}\right)\right|}{N},\;\;N=\frac{n\left(n-1\right)}{2}.$$

Where cor(*p*_i_, *p*_j_) denotes the Pearson correlation coefficients of proteins *p*_i _and *p*_j_.

## Results

### Comparison on breast cancer datasets GSE10797 and GSE7904 for a two-class case

**Figure 2:**
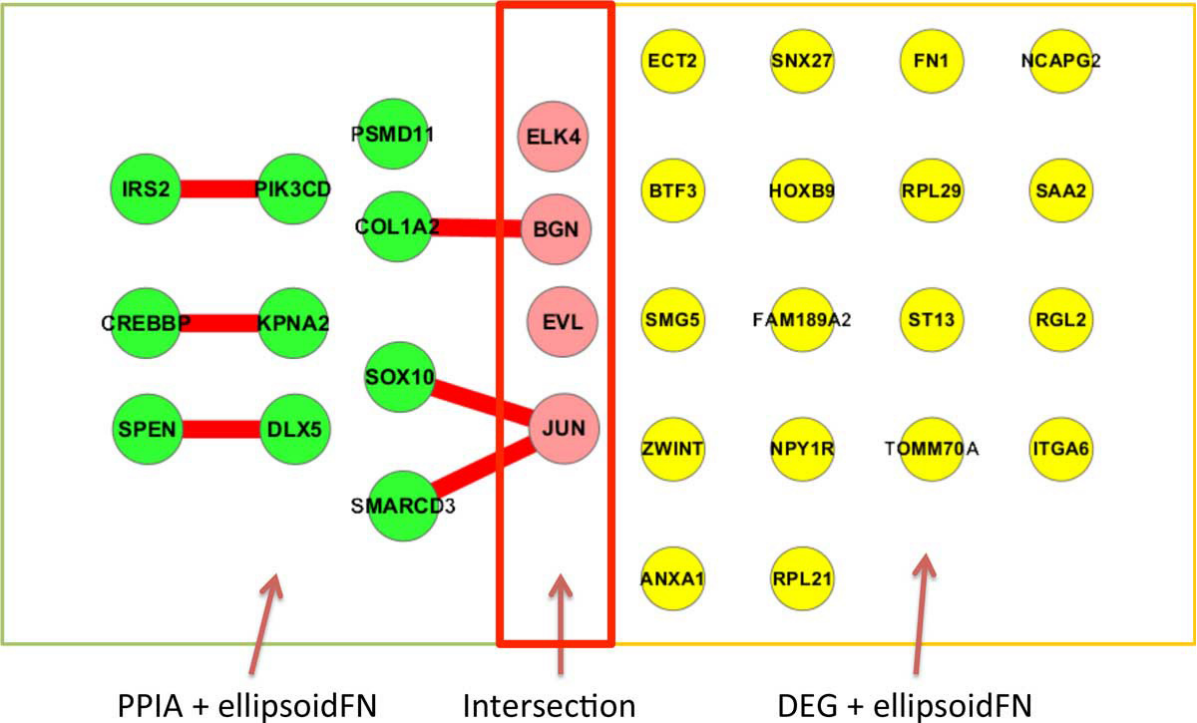
**Biomarkers identified by PPIA + ellipsoidFN and DEG + ellipsoidFN for the breast cancer data GSE10797**. Network biomarkers identified by PPIA + ellipsoidFN contain 3 proteins and 6 protein-protein interactions including 14 genes, while DEG + ellipsoidFN identified 22 genes. 4 genes are in common for the two methods.

There are totally 22,277 probes and 66 samples including 10 normal samples (5 normal stromal samples and 5 normal epithelial samples) and 56 disease ones (28 stromal samples of breast cancer and 28 epithelial samples of breast cancer) in the breast cancer dataset GSE10797. Genes with missing values and with low information were filtered out from the raw data. Here low information means the entropy of genes expression distribution is smaller than 1.5. After these procedures, we got a gene set with 2,325 genes. Then we mapped the probes to human PPI network and 5,458 gene pairs were obtained. Based on our PPIA + ellipsoidFN optimization model and Random Forest classifier (we use the MATLAB toolbox with version 0.02 and license GPLv2), 3 genes and 6 interactions totally including 14 genes (Supplementary Data Set 1) were identified with leave-one-out cross-validation classification accuracy 96.97% (64/66), while DEG + ellipsoidFN method got 22 genes (Supplementary Data Set 2) with classification accuracy 93.94% (62/66). The overlap of these two sets of biomarkers contains 4 genes. Figure 2 demonstrates the biomarkers identified by PPIA + ellipsoidFN and DEG + ellipsoidFN for the breast cancer data GSE10797. The PPIA + ellipsoidFN method got higher accuracy than DEG + ellipsoidFN. Since we simultaneously selected nodes and edges, the better performance of classification results from the network information added to the original gene expression data. Clearly, the protein-protein interaction biomarkers identified by our method include fewer genes, and those interactions also have clear biological functions related to pathways. The mean redundancy score of PPIA + ellipsoidFN method is 0.1839 which is lower than that of DEG + ellipsoidFN 0.2090. This result demonstrates the non-redundant property of the network biomarkers including genes and PPIs. Table 1 shows the detailed information for comparison.

**Table 1:** Performance comparison among various methods.

A					
	**Data sets**	**Method**	**Predicting accuracy**	**Redundancyscore**	**Number of genes**

Two-class case	GSE10797	DEG+ellipsoidFN	93.94% (62/66)	0.2090	22
		
		**PPIA+ellipsoidFN**	**96.97% (64/66)**	**0.1839**	**14**
	
	GSE7904	DEG+ellipsoidFN	98.39% (61/62)	0.3698	18
		
		**PPIA+ellipsoidFN**	**98.39% (61/62)**	**0.2968**	**19**

Multiple class	GSE18229	DEG+ellipsoidFN	80.00% (248/310)	0.1532	170
		
		**PPIA+ellipsoidFN**	**79.03% (245/310)**	**0.1652**	**135**
	
	GSE10797	DEG+ellipsoidFN	78.79% (52/66)	0.1663	161
		
		**PPIA+ellipsoidFN**	**83.33% (55/66)**	**0.2150**	**56**

**B**					

	**Data sets**	**Method**	**Redundancy score**	**Number of common genes**	

Two-class case	GSE10797	t-test	0.2715	13	
				
		**PPIA**	**0.2144**		
	
	GSE7904	t-test	0.3077	27	
				
		**PPIA**	**0.2623**		

Multiple class	GSE18229	F-test	0.3031	15	
				
		**PPIA**	**0.1693**		
	
	GSE10797	F-test	0.3280	11	
				
		**PPIA**	**0.2209**		

(A) Comparing PPIA + ellipsoidFN method with DEG + ellipsoidFN in predicting accuracy, redundancy score, and the number of genes identified. (B) Comparisons on PPIA + ellipsoidFN and t-test for two-class case and PPIA + ellipsoidFN versus F-test for multiple-class case based on 50 top PPIs and proteins.

Also it is important to note that when we reduce the total number of genes from 2,325 (including 5,484 PPIs) to 935 (containing 1,527 PPIs) by entropy distribution, our PPIA + ellipsoidFN method gained a predicting accuracy 93.94% compared to that of DEG + ellipsoidFN 84.85%. This result shows when fewer genes and PPIs are used for initial selection, network biomarkers are robust and superior to traditional single molecules. One reason is that strictly selected genes reduce noise of the data set. Generally, in a molecular network, genes work as a system and interact with each other to achieve certain functions.

In order to compare the performance of t-test and PPIA + ellipsoidFN in a fair way, we selected top 50 network biomarkers including both PPIs and proteins and compared their redundancy scores. PPIA + ellipsoidFN got a redundancy score 0.2144 versus 0.2715 of t-test, and the number of the intersection of these two biomarker sets is 13. These results show that our PPIA + ellipsoidFN method can identify a set of heterogeneous network biomarkers which are considerably different from the widely used t-test. Comparisons on t-test and PPIA + ellipsoidFN are demonstrated in Table 1.

In order to strengthen the advantage of our method, we take another data set GSE7904 for further evaluation. This breast cancer data contains totally 54,675 probes and 62 samples, including 19 normal samples and 43 disease ones. After processing the data with the method above, 516 genes including 682 PPIs left. The leave-one-out cross-validation classification accuracy is 98.39% (61/62) for both PPIA + ellipsoidFN and DEG + ellipsoidFN methods. Also 2 genes and 9 interactions containing 19 genes (Supplementary Dataset 3) were selected as network biomarkers with the redundancy score 0.2968, while 18 genes (Supplementary Dataset 4) were identified by DEG + ellipsoidFN method with the redundancy score 0.3698. Both biomarker sets share 4 genes in common. This computational experiment shows that the two methods achieve the same classifying accuracy but network biomarkers reduce the redundancy score of the biomarker set. The comparison results are presented in Table 1. The performance of t-test and PPIA + ellipsoidFN were compared in a similar way (choose top 50 PPIs and proteins for comparison), for detailed results please refer to Table 1.

### Comparison on datasets GSE18229 and GSE10797 for multiple-class case

Dataset GSE18229 contains 337 samples and 22,575 probes in total. Filtering the samples without subtype label and normal ones, we got 310 tumor samples including 73 basal-like, 37 claudin, 39 Her2, 99 luminal A, and 62 luminal B. The procedures of pre-preprocessing data are similar to those above in two-class case. Experimental results show that the predicting accuracy is 80% for DEG + ellipsoidFN and 79.03% for PPIA + ellipsoidFN method. Similarly the redundancy score is 0.1523 out of 170 genes (in Supplementary Dataset 6) for DEG + ellipsoidFN, and 0.1652 out of 90 genes and 38 interactions which contains 135 genes (in Supplementary Dataset 5) for PPIA + ellipsoidFN method. The overlap of these two sets of biomarkers contains 94 genes. This result illustrates that the predicting accuracy of PPIA + ellipsoidFN is slightly lower than that of DEG + ellipsoidFN. The result of the redundancy score could arise from the fact that interacting proteins are inclined to be co-expressed, which is different from gene-node biomarkers. These comparisons could also be checked in Table 1.

We also introduce dataset GSE10797 for multiple-class case. PPIA + ellipsoidFN method identifies 12 genes and 23 PPIs including 55 genes (Supplementary Dataset 7) as network biomarker with the redundancy score 0.2150 and the classifying accuracy is 83.33% (55/66). DEG + ellipsoidFN method achieves the classifying accuracy 78.79% (52/66) and identifies 161 genes (Supplementary Dataset 8) as node marker with the redundancy score 0.1663. The intersection of the two biomarker sets contains 21 genes.

For data set GSE18229 PPIA + ellipsoidFN method exploiting 50 top PPIs and proteins share 15 proteins in common with F-test, and the redundancy scores are 0.3031 for F-test and 0.1693 for PPIA + ellipsoidFN. For data set GSE10797 with four-class situation, PPIA + ellipsoidFN method shares 11 PPIs and proteins in commons with F-test, and the redundancy scores are 0.2209 for PPIA + ellipsoidFN method versus 0.3280 for F-test. The results are shown in Table 1 and demonstrate the heterogeneous property of biomarkers identified by the PPIA + ellipsoidFN method.

### PPI biomarkers identified by PPIA + ellipsoidFN method for two breast cancer data set

**Figure 3:**
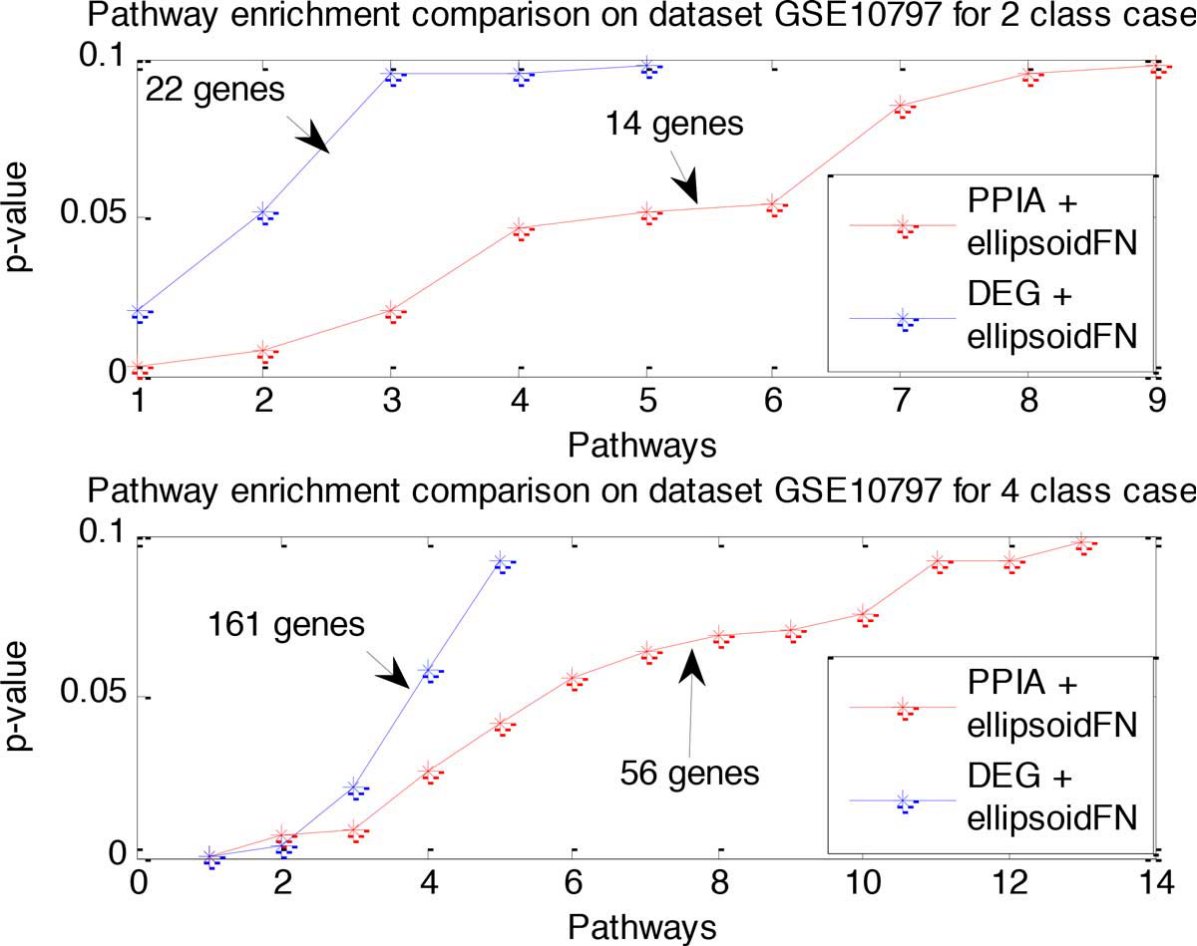
**Comparison on pathways enriched by biomarkers identified from PPIA + ellipsoidFN and DEG + ellipsoidFN method**. For two biomarker sets identified by the two methods respectively, we used KEGG pathway enrichment search to find several pathways according to their p-values. Then we sort these p-values in ascending order and make a comparison between the two methods on dataset GSE10797 for both two-class and four-class cases. X-axis denotes pathways, while y-axis denotes the p-value of each corresponding pathway. For example, the blue line in the figure on the top shows there are five pathways in total enriched by the node biomarkers whose p-value is lower than 0.1. The small number of pathways that are significantly enriched may result from the fact that there are few genes in the biomarker set and their non-redundant property. From the figure we can easily see that network biomarkers identified by PPIA + ellipsoidFN method tends to enriched to more pathways with lower p-value.

We obtained 9 PPIAs to optimally classify the normal and breast cancer samples with PPIA + ellipsoidFN on data set GSE10797 for two-class situation. These genes and pairs are PSMD11, ELK4, EVL, SPEN-DLX5, CREBBP-KPNA2, BGN-COL1A2, SMARCD3-JUN, IRS2-PIK3CD, and SOX10-JUN. In total, 14 genes are involved in those interactions. Evidence from a literature search implies that some of the node and interaction biomarkers above are involved in breast cancer. Lower EVL expression correlates with high invasiveness and poor patient outcome in human breast cancer [16], and the expression of the EVL protein is significantly expressed in breast cancer-derived MCF7 cells [17]. Moreover, PSMD11 was over-expressed in breast cancer tissue compared to adjacent normal tissue [18]. SOX10, MITF, and JUN were significantly regulated in melanomas in comparison with cancer [19]. BGN, COL1A1, COL1A2, MMP9, CD44, FN1, TGFBI, PXN, SPARC, and VWF were associated with tumor metastasis and formed a highly interactive network with the first four molecules as hubs [20]. Exploiting DAVID functional annotation tool [21,22] and KEGG pathway analysis [23], the 14 genes are functionally enriched in several pathways and biological process with statistical significance. Among them, HDAC4-mediated deacetylation of the SMAD4 promoter may lead to 5-FU resistance in breast cancer cells [24]. Five significant pathways including focal adhesion are discovered in breast cancer metastasis [25]. The comparison on KEGG pathway enrichment of PPIA + ellipsoidFN and DEG + ellipsoidFN methods based on p-value is shown in Figure 3, which demonstratesthat our PPIA + ellipsoidFN method tends to identify biomarkers which are more enriched to functional pathways. This result arises from the fact that network biomarkers identified by our method include more interaction information rather than the single node selection method. Moreover, Pathways obtained by KEGG pathway search and functional analysis from DAVID for breast cancer data GSE10797 are listed in Table 2. This table also shows the corresponding result of DEG + ellipsoidFN method. From the table, we can find that biomarkers identified by both methods are enriched to Focal adhesion and Pathways in cancer, which are two pathways greatly related to breast cancer.

**Table 2:** KEGG pathway and DAVID functional analysis results for PPIA + ellipsoidFN method and DEG + ellipsoidFN method on data set GSE10797 for two class case.

	PPIA + ellipsoidFN	DEG + ellipsoidFN
KEGG pathways	Renal cell carcinoma Neurotrophin signaling pathway Focal adhesion Aldosterone-regulated sodium reabsorption Pathways in cancer	Focal adhesion Pathways in cancer Small cell lung cancer ECM-receptor interaction Ribosome

DAVID functional analysis	GOTERM_MF_FAT transcription factor binding GOTERM_MF_FAT transcription cofactor activity RT GOTERM_MF_FAT SMAD binding GOTERM_BP_FAT positive regulation of macromolecule metabolic process	GOTERM_BP_FAT peptide cross-linking GOTERM_CC_FAT cytosol SP_PIR_KEYWORDS phosphoprotein UP_SEQ_FEATURE cross-link:Isoglutamyl lysine isopeptide (Gln-Lys) GOTERM_MF_FAT structural molecule activity

The top 5 terms are listed and ranked by their p-values.

2 genes and 9 protein-protein interactions including 19 genes were identified as network biomarkers with our PPIA + ellipsoidFN method on GSE7904 data set. We studied their relationships with breast cancer by applying the DAVID functional annotation tool. This set of genes enriches phosphoprotein with p-value 1.2E-5. From a literature search, there is evidence that some phosphoproteins have the potential to qualify as phosphopeptide plasma biomarker candidates for the more aggressive basal and also the luminal-type breast cancers [26]. Tumors of the Triple negative breast cancer (TNBC) subtype showed high deregulation of many proteins and phosphoproteins [27].

Moreover, for dataset GSE10797 in a four-class case, we also found some evidence with the help of KEGG pathway search, we found that several pathways are statistically significant, e.g., hsadd05200 Pathways in cancer, hsa04012 ErbB signaling pathway and hsadd04510 Focal adhesion pathway. ErbB-2 is a target for cancer-initiating cells in breast and other cancers [28]. Amplification and subsequent overexpression of the HER2 encoding oncogene results in unregulated cell proliferation in HER2-positive breast cancer [29]. All the evidence above demonstrates that the network biomarkers identified by our PPIA + ellpsoidFN method have efficiency and comprehensibility from a biological perspective.

### Comparison with another approximation method for protein-protein interaction

In this paper we make an estimation of protein-protein interaction affinity derived from the law of mass action, i.e., *a*_*ij *_= *x*_*iu *_*x*_*iv *_There is another way to measure the co-expression pattern of the edges, which is defined by the absolute value of the difference between the two proteins, i.e., *a_ij _*= |*x_iu _*- *x_iv_*|. We compared the two methods by their classification accuracy (10-fold cross-validation with Random Forest classifier). For two-class case, the differential form gives out the predicting accuracy 93.94% (62/66), while the product-form gains 96.97% (64/66) on dataset GSE10797. And for dataset GSE7904 the two methods both achieve the same accuracy of 98.39%. For multi-class case, the differential form achieves 76.45% versus 79.03% for the product-form on data set GSE18229. And on data set GSE10797 for four-class case, the differential form gives 75.76%, while the product-form gains 83.33%. These results show that our PPIA estimation derived from the law of mass action performs better than the differential form from classification view.

### Comparison with existing edge biomarker selection method

In this study we propose a linear programming based optimization model to find the optimal set of protein-protein interaction affinities which are measured by the product of the gene expression. The physical meaning of PPIA is clear based on the law of mass action. Then, the product of the protein pairs in terms of concentration is the approximation for the activity of protein-protein interaction in the cell, which is a similar measure as the correlation based measure. In this paper, we also compared the performance of product approximation with the measure of EdgeMarker [6] (defined by the decomposition of PCC) with Naïve Bayesian classifier by predicting accuracy in our optimization model. Computational result shows that the decomposed PCC used in EdgeMarker got 84.85% correct compared to 93.94% of product approximation when running on the breast cancer data GSE10797, and 42.6% correct of the decomposed PCC compared to 70.65% of product approximation when running on the breast cancer data GSE18229. These results can further illustrate the advantages and efficiency of our PPIA-based method.

### The importance of dimension reduction in our method

If we don't use the ellipsoidFN method to reduce the dimension of the data, the classification accuracy will be much lower. Now we only apply the Random Forest classifier to make classification and prediction. For two-class case on data set GSE10797, random Forest could only achieve 84.89% accuracy, and on data set GSE18229 the accuracy is 63.17%. Moreover, the program needs much longer running time. This comparison could demonstrate the efficiency of our dimension reduction model ellipsoidFN.

## Discussions

The complexity of cancer pathogenesis makes it difficult to identify a set of effective and stable biomarkers for the diagnosis of complex diseases. In this study, we incorporate PPI with gene expression data to battle cancer heterogeneity. Because PPIs bring the topological information of a network, our method has clear advantages over the node based feature selection method. Generally, our method tends to select pathways or subnetworks, which have clear and enriched biological meanings. Our main contribution in this work is to integrate PPI with expression data in a novel way by defining PPIA, which has clearer biochemical meaning. Moreover, ellipsoidFN, an efficient dimension reduction method to select a representative subset of PPIA, is utilized to perform the feature selection from high dimensional data. With the increasing biological interpretability, the redundancy score of biomarkers identified by our method increases in some cases and the predicting accuracy is comparable to DEG + ellipsoidFN. Those results also demonstrate the tradeoff between the biological interpretation and machine learning accuracy.

A network is a set of edges or interactions. Those interactions are context-specific and dynamic in nature. Under different conditions, different interactions are selectively activated or deactivated. Importantly, network rewiring is a general principle of biological systems regarding disease progression or therapeutic response in epigenomic era. In this paper, we propose a new method to combine PPIA and ellipsoidFN to detect network biomarkers from pathway perspective rather than single node based identification methods. We stress "which interactions are differentially connected" instead of "which genes are differentially expressed".

As we mentioned, this edge-focused study provides a general data integration framework for static network information and dynamic expression data. We note that there are different ways to consider edge information. Liu et al. [30] integrated the information of differential expression status of gene and co-expressed gene pair to form a scoring scheme based on Fisher's method. This scoring scheme helps to construct a weighted PPI network which was used to detect differentially activated pathways [31] and identify dysfunctional crosstalk of pathways in different regions of Alzheimer's disease brain [30]. Recently, Sun et al. [32] provided a novel way to combine the two data sources. They identified differential and non-differential interactions, having no significant change on edge strength but the linked two genes are both differentially expressed, to study type 2 diabetes. The methods above to combine the gene expression profiling and PPI network data could be utilized to construct various types of differential network. And the ellipsoidFN model can be built on these networks and detect new sets of network biomarkers, which have different physical meanings and biological functions for certain type of diseases. Another possible promising improvement is to consider the relationships between different phenotypes, such as tumor stages, to identify phenotype responsive biomarkers. It is also interesting to further move from interaction to module level, which identifies module biomarkers [33-35] in biological networks. It is also important to consider the dynamical information for predicting early state of complex diseases, by dynamical network biomarkers [36-38] and edge biomarkers [39].

## Competing interests

The authors declare that they have no competing interests.

## Supplementary Material

Additional file 1 **Supplementary Dataset 1**.txt Click here for file

Additional file 2 **Supplementary Dataset 2**.txt Click here for file

Additional file 3 **Supplementary Dataset 3**.txt Click here for file

Additional file 4 **Supplementary Dataset 4**.txt Click here for file

Additional file 5 **Supplementary Dataset 5**.txt Click here for file

Additional file 6 **Supplementary Dataset 6**.txt Click here for file

Additional file 7 **Supplementary Dataset 7**.txt Click here for file

Additional file 8 **Supplementary Dataset 8**.txt Click here for file

Additional file 9 Source code for the new method Click here for file
